# Research on the Mechanical Model and Hysteresis Performance of a New Mild Steel-Rotational Friction Hybrid Self-Centering Damper

**DOI:** 10.3390/ma16227168

**Published:** 2023-11-15

**Authors:** Debin Wang, Ran Pang, Gang Wang, Guoxi Fan

**Affiliations:** 1 School of Civil Engineering, Dalian Jiaotong University, Dalian 116028, China; wdb1215@djtu.edu.cn (D.W.); pran0716@163.com (R.P.); 2 School of Engineering, Ocean University of China, Qingdao 266100, China

**Keywords:** self-centering, restoring-force model, hysteresis curve, energy dissipation, residual deformation

## Abstract

A mild steel-friction self-centering damper with a hybrid energy-dissipation mechanism (MS-SCFD) was proposed, which consisted of a mild steel, frictional, dual-energy-dissipation system and a disc spring resetting system. The structure and principle of the MS-SCFD were explained in detail while the restoring force model was established. The hysteretic behavior of the MS-SCFD under low-cycle reciprocating loading was modeled. Then, the influence of parameters such as the disc spring preload, the friction coefficient, and the soft-steel thickness on the mechanical properties of the MS-SCFD was investigated. The results indicate that the simulation results are basically consistent with the theoretical prediction results, with a maximum error of only 9.46% for the key points of bearing capacity. Since the MS-SCFD is provided with a hysteretic curve in the typical flag type, it will obtain the capacity of excellent self-centering performance. It can effectively enhance the stiffness, bearing capacity, and self-centering capability of the damper after the pre-pressure of the disc spring is increased. The energy-dissipation capacity of the MS-SCFD increases with the increase in the friction coefficient. However, it also increases the residual deformation of the MS-SCFD. The energy dissipation of the MS-SCFD is particularly sensitive to the thickness of mild steel. After being loaded, all components of the MS-SCFD are not damaged except for the plastic deformation caused by the yielding of the mild steel. The normal function of the MS-SCFD can be restored simply by replacing the mild steel plates after the earthquake. Therefore, it can significantly enhance the economy and applicability of the damper.

## 1. Introduction

Currently, depending on the working mechanisms, energy-dissipation devices in seismic engineering structures are commonly categorized into friction dampers [1,2,3,4,5,6], metal yield dampers [7,8,9,10,11], viscoelastic dampers [12,13,14], viscous liquid dampers [15,16,17], etc. Traditional dampers usually adopt a particular energy-dissipation mode, most of which lacks a self-centering ability, resulting in the structure of the attached damper being damaged after the earthquake and producing plastic deformation, which is difficult to recover. Therefore, various mechanical structures have been designed to empower energy-dissipation devices with a self-centering functionality. Besides the vibration control of structures, these devices can also strengthen existing buildings.

Mild steel is extensively adopted in metal self-centering dampers, attributing to its exceptional resistance against low cycle fatigue, high elongation, and pronounced plastic deformation capability due to its low yield strength, relative stability, and low yield ratio. After achieving the plastic state, this steel exhibits excellent hysteretic performance and effectively absorbs substantial amounts of energy during the process of elasto-plastic hysteresis deformation [18,19]. The SCEDB-U, a self-centering brace proposed by Jia et al. [20], exhibits negligible degradation in stiffness and strength, demonstrates stable energy-dissipation performance, achieves minimal residual deformation, and shows excellent self-centering capabilities under low cyclic protocols reciprocating loads. Naeem et al. [21] proposed a self-centering disc slit damper (SC-DSD). The energy-dissipation function of this damper was realized by the yielding of an external hollow steel strip in a box, and the restored force was provided by the embedded composite disc springs. The test results showed that the damper had a stable flag-shaped hysteretic behavior and minimal residual deformation, and the bearing capacity and self-centering capacity of the damper were significantly improved compared with the traditional steel slit damper. Although the yielding-based energy-dissipating type self-centering metallic dampers have the advantages of strong energy-dissipation capacity, diversified structural forms, and low cost, they can only provide lateral stiffness at a low displacement level. In other words, this type of damper can only generate energy dissipation under severe earthquakes [22]. Therefore, many scholars prefer using friction as the energy-dissipation mechanism of self-centering dampers to meet the demand for energy absorption at a low displacement level. Xu et al. [23] developed an assembled pre-pressed spring self-centering energy-dissipation damper (A-PS-SCED), which used disc springs to provide restored forces and core plate friction for energy dissipation. The simulation results indicated that the damper is stable and has plump hysteretic behavior. It operates well at a low displacement level with nearly zero residual deformation. Wang et al. [24] proposed a self-centering damper with a displacement-amplification rotation friction function (SC-DARFB) based on the principle of bridge mechanism amplification. The initial amplification angle can improve the energy-dissipation capacity of the brace at a low displacement level. The displacement at the top of the pier and the residual displacement of the damper could be effectively controlled when the SC-DARFB was attached to the double-column pier structure. Wang et al. [25] introduced a variable friction mechanism and proposed the SC-VFD. This device exhibited a smaller residual displacement than the conventional VFD while maintaining the same energy-dissipation capacity. However, it has a significantly lower equivalent viscous damping ratio. Most metal-yielding or friction-type self-centering dampers are either tensional or compressional energy-dissipation dampers, which often have problems such as a single energy-dissipation mechanism and excessive consumption of combined disc springs.

Hybrid dampers, incorporating diverse energy-dissipation mechanisms, usually exhibit enhanced efficiency in dissipating energy compared to the conventional single-mechanism damper. The self-centering damper proposed by Qiu et al. [26] incorporates shape memory alloy (SMA) and a steel plate to achieve dual energy dissipation, contributing to a simple structure with excellent centering performance and a high damping ratio. Lu et al. [27] proposed a friction-steel strip hybrid energy-dissipation damper (FSCD). They conclude from the test results that the energy-dissipation process can be divided into three stages: the synergistic action, the metal part fracture, and the pure friction stage. They also observed that the damper’s strength, stiffness, and energy dissipation increase with the increase in friction force and the width of the steel strip. Gao et al. [28] proposed an assembled buckling sleeve with a friction damper (FSM). The test showed that, under the same axial small-displacement loading, the FSM had greater damping force and energy-dissipation capacity than the ordinary assembled sleeve member (ASM). Lu et al. [29] proposed a new type of buckling-restrained damper (BFD) with an additional frictional energy-dissipation mechanism. The test shows that the damper can achieve multi-stage yield energy dissipation, and the additional frictional energy-dissipation mechanism effectively enhances the energy-dissipation capacity of the damper. Ke et al. [30] combined mild steel with a frictional energy-dissipation mechanism and proposed a displacement-type hybrid damper with multiple yield stages, which exhibits good ductility and an expected energy-dissipation sequence. All the aforementioned hybrid dampers have two energy-dissipation elements. Both mechanisms complement each other, maximizing the dampers’ function and efficiency and meeting the structures’ multiple seismic requirements. However, insufficient attention has been paid to the self-centering performance of the dampers, most of which are tensional or compressional dampers.

Based on the above analysis, a mild-steel self-centering friction damper (MS-SCFD) with a hybrid energy-dissipation mechanism is proposed in this paper. Firstly, the basic structure and the working mechanism of the MS-SCFD were introduced, and its theoretical restoring force model was derived. Then, the practical engineering applications of the MS-SCFD were introduced, and the numerical simulation model was established to verify the theoretical restoring-force model. Finally, the mechanical properties of the new damper are investigated. Dampers with different parameters, such as initial pre-pressure, friction coefficients, and mild steel thicknesses, are comprehensively analyzed to explore the applicability of the damper.

## 2. Basic Structure and Working Principle of MS-SCFD

### 2.1. Structure of MS-SCFD

**Figure 1:**
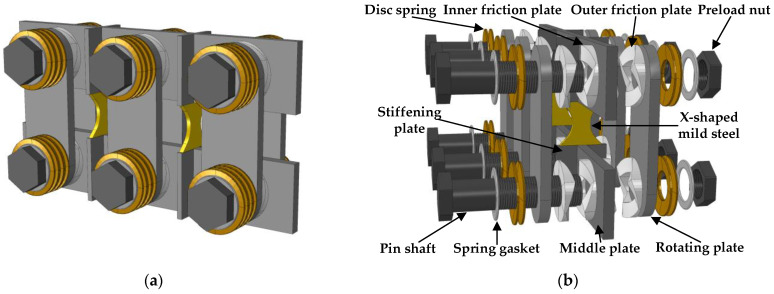
Overall and disassembly diagram of the MS-SCFD. (**a**) General diagram of the MS-SCFD structure; (**b**) schematic diagram of dismantling the MS-SCFD.

The damper primarily consists of a self-centering friction energy-dissipation system and a metallic yield energy-dissipation system. Its specific construction is depicted in Figure 1. The self-centering friction energy-dissipation system comprises the inner friction plates fixed on the middle plate, the outer friction plates fixed on the rotating plates, the pin shafts, the combined disc springs, the preload nuts, and the spring gaskets. The metal yield energy-dissipation system comprises a detachable X-shaped energy-dissipation mild steel and a stiffening plate for fixed mild steel.

The MS-SCFD was designed by adding a mild-steel energy-dissipation enhancement device to the self-centering friction damper (SCFD) while maintaining the characteristics of the SCFD. In the self-centering friction energy-dissipation system, the inner and outer friction plates are equipped with six spiral surfaces, and the adjacent surfaces are arranged alternately. The X-shaped mild steel and the friction energy-dissipation system are arranged alternately, and the middle plate is fixed and connected with stiffening plates arranged outside the plane of the middle plate. The combination of disc springs in the self-centering system is symmetrically set at both ends of the pin shaft and is closely fitted with the rotating plate. A spring gasket is also positioned between the pin shaft and disc springs. Overlap or composite stacking arrangements, including single-layer and multi-layer, can be selected according to the design requirements of the disc springs. Only a limited quantity of disc springs meets the requirements for damping characteristics and self-centering performance, thereby eliminating the need for traditional self-centering dampers for numerous disc springs to accommodate significant large deformation.

### 2.2. Working Mechanism

The MS-SCFD system operates in two energy-dissipation modes, specifically, friction energy dissipation for the extrusion of inner and outer friction plates and bending yield energy dissipation for X-shaped mild steel. The combined disc springs provide normal pre-pressure to restore the damper’s two energy-dissipation systems completely.

**Figure 2:**
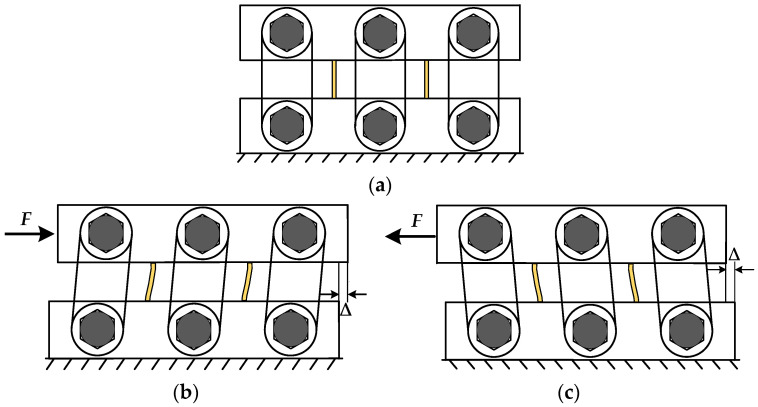
Working mechanism of the MS-SCFD. (**a**) Initial state; (**b**) state of compression; and (**c**) state of tension.

In this paper, based on the seismic design code of buildings [31] and the characteristics of the inter-story shear deformation applied to multi-high-rise eccentric brace steel frame structures, the methods of [32] are used as the design basis to elaborate the working mechanism of the damper as shown in Figure 2. Before the loading of the MS-SCFD, the spiral surfaces of the inner and outer friction plates are seamlessly and tightly engaged. First, the initial pre-pressure is applied to the combined disc springs following the design requirements to provide the restored force for the MS-SCFD. When the load is applied to the middle plate at the top of the MS-SCFD, the inner friction plate at the bottom is in a static state. The inner friction plates at the top will overcome the static friction moment and produce translation under the action of external load. The outer friction plates will rotate around the pin shaft in relation to the inner friction plate, subsequently leading to a mutual compression between the inner and outer friction plates. This process results in energy dissipation through friction under the pre-pressure exerted by the combined disc springs. Meanwhile, the lower part of the outer friction plate also undergoes rotational motion around the fixed pin shaft, resulting in energy dissipation through friction. With the continuous increase in load, the compression deformation of the combination of disc springs increases, and the normal side pressure also increases, which increases the friction moment of the friction contact surface. The lower end of the X-shaped mild steel is in a fixed constraint state, while the upper middle plate drives the upper end to induce synchronous displacement, out-of-plane bending, shear deformation, and yield energy dissipation. The inner and outer friction plates rotate in reverse when unloading. The compression deformation of the combined disc springs gradually decreases and returns to the initial equilibrium position. At the same time, since the friction energy-dissipation system can be restored to the initial position without restored force, the restored force provided by the pre-pressed composite disc springs will be used to eliminate the plastic deformation generated by the X-shaped mild steel to achieve the MS-SCFD reset.

### 2.3. Practical Engineering Application

**Figure 3:**
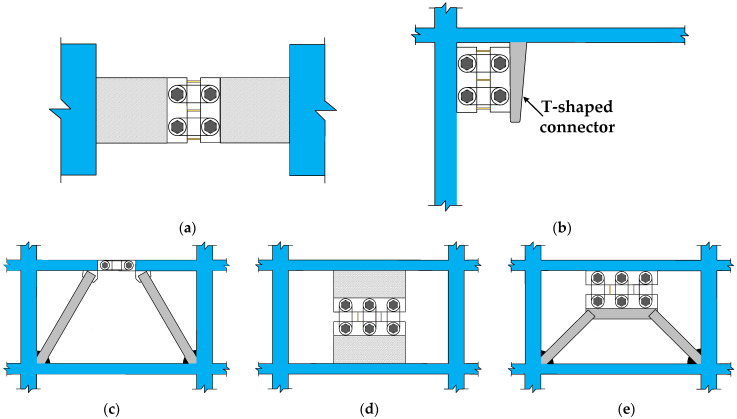
Examples of MS-SCFD engineering applications. (**a**) Shear wall with connected beam; (**b**) beam–column joint; (**c**) eccentrically k-braced steel frame; (**d**) interstory self-centering shear wall; and (**e**) herringbone braced frame.

The MS-SCFD proposed in this paper exhibits broad engineering applicability and can be effectively deployed in various structural positions. For instance, in the connected beam part of the shear wall structure, relative displacement occurs at both ends of the connected beam, which drives the shear deformation of the damper to achieve its reset and energy-dissipation performance, as shown in Figure 3a. In addition, the damper can also be deployed at positions such as the corner of the beam and column joints (Figure 3b), the K-shaped eccentric brace frame (Figure 3c), the shear wall between the upper and lower wall (Figure 3d), the herringbone brace frames (Figure 3e), and other positions. The MS-SCFD plays a crucial role in the aforementioned applications by effectively absorbing and dissipating seismic energy during earthquakes and providing a restored force to prevent failure and excessive plastic deformation of the structural elements. When the MS-SCFD is damaged during an earthquake, the X-shaped mild steel can be quickly replaced without significantly affecting the normal function of the main structure.

## 3. Theoretical Model of the MS-SCFD

### 3.1. Restoring Force Model of the SCFD

**Figure 4:**
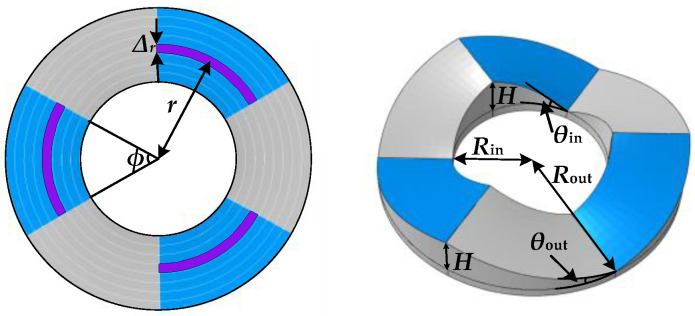
Geometric parameters of the friction plate.

The parameters of the inner and outer friction plates of the self-centering friction energy-dissipation system are shown in Figure 4. The inclination angle of its spiral surface *θ* gradually decreases from inside to outside (*θ*_out_ < *θ*_in_). The general value range of *θ*_out_ in practical applications is between 12° and 30°, and this article takes *θ*_out_ = 15°. *H*, *R*_out_, and *R*_in_ are the friction plate height, outer edge radius, and shaft hole radius_,_ respectively. ∆*_r_* is the width of a single friction strip. *ϕ* represents the projection angle of the spiral surface; as specified in this article, the value of *ϕ* is 60°. Only three groups of friction surfaces contact and dissipate energy at each friction energy-dissipation part during loading.

The loading and unloading friction moment *M* yields the sum of the static friction moments produced by the six helical surfaces of the two groups of friction contact surfaces. According to the literature [33], the computation equations for *M* during loading and unloading are as follows:

(1) $$M=\frac{2P}{R_{\mathrm{out}}-R_{\mathrm{in}}}\int_{R_{\mathrm{in}}}^{R_{\mathrm{out}}}\left(\frac{H+\mu_{s}\phi r}{\phi r-\mu_{s}H}\right)rdr$$

(2) $$M=\frac{2P}{R_{\mathrm{out}}-R_{\mathrm{in}}}\int_{R_{\mathrm{in}}}^{R_{\mathrm{out}}}\left(\frac{H-\mu_{k}\phi r}{\phi r+\mu_{k}H}\right)rdr$$

where *P* stands for the combined disc spring pre-pressure in the formula, and *μ*_s_ and *μ*_k_ stand for the loading and unloading friction coefficients, respectively, with the same values in this study.

**Figure 5:**
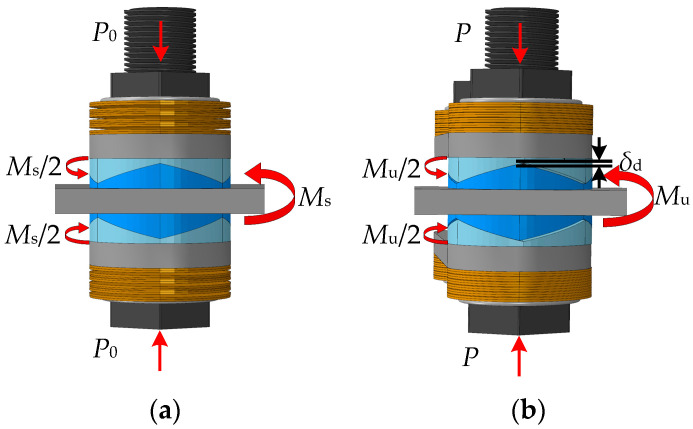

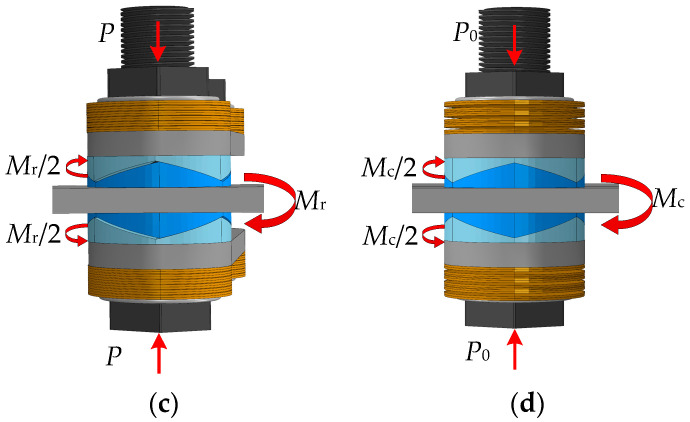
Vertical view of the SCFD (partial) loading. (**a**) Slip moment *M*_s_; (**b**) ultimate moment *M*_u_; (**c**) ultimate unloading moment *M*_r_; and (**d**) restored moment *M*_c_.

The rotation deformation and friction moment of the four key performance points produced by a single set of friction plates during the SCFD working are shown in Figure 5. The slip moment *M*_s_, and the ultimate moment *M*_u_, are calculated using Equation (1). The ultimate unloading moment, *M*_r,_ and the restored moment, *M*_c,_ during unloading are calculated using Equation (2). Note that the calculation process only requires the modification of the pre-pressure *P* of the combined disc springs under different compression deformation, and its calculation expression is given in Equation (3):

(3) $$P=P_{0}+k_{d}\delta$$

where *k*_d_ is the stiffness of the combined disc springs, and *δ* is the compression amount of the combined disc springs under different displacement amplitudes.

**Figure 6:**
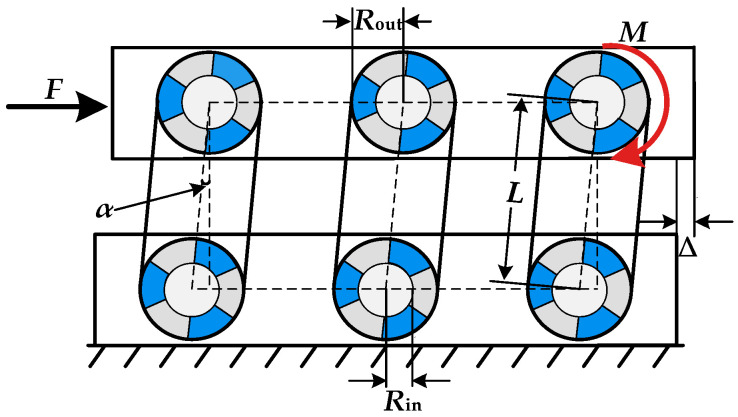
Front view of the SCFD loading.

The relative rotation angle and displacement variations of the inner and outer friction plates throughout the SCFD loading process are schematically depicted in Figure 6. Under small deformation conditions, it is evident that the lateral displacement and rotation angle approximate one another as follows:

(4) Δ=αL

where *L* is the effective length of the rotating plate. It can be seen from Figure 5 that there is the following relationship between the compression amount *δ* of the combination of the disc springs under the load of the SCFD and the relative angle *α* of the inner and outer friction plates:

(5) $$\frac{\delta }{\tan\theta_{\mathrm{out}}}=R_{\mathrm{out}}\alpha$$

Therefore, the following relationship exists between the compression *δ* of the combined disc springs and the lateral displacement ∆ of the SCFD by substituting Equation (4) into Equation (5):

(6) $$\delta =\frac{R_{\mathrm{out}}\tan\theta_{\mathrm{out}}}{L}\Delta$$

During the loading process, the upper load *F* of the SCFD equals the sum of the friction moments of the six groups of friction plates so that the relationship between the theoretical value *F* of the output force of the damper and the friction moment *M* can be computed as follows:

(7) $$F=\frac{6M}{L\cos\alpha }$$

**Figure 7:**
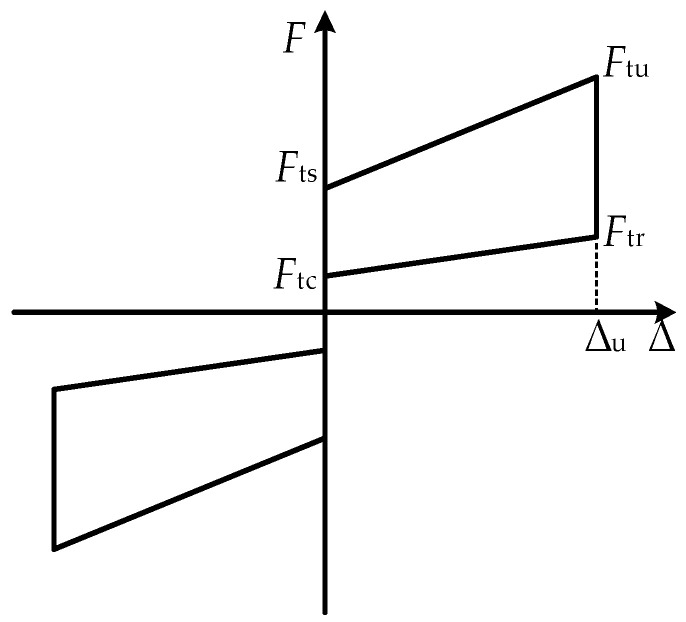
Theoretical restoring-force model of the SCFD.

Based on the above analysis, the force values of the key performance points in different loading and unloading stages of the SCFD can be obtained through calculation, where *F*_ts_, *F*_tu_, *F*_tr,_ and *F*_tc_ are the slip force, the ultimate force, the ultimate unloading force, and the restored force, respectively. The hysteresis curves of the SCFD theoretical restoring-force model were obtained, as shown in Figure 7.

### 3.2. Restoring-Force Model of the X-Shaped MS Damper

**Figure 8:**
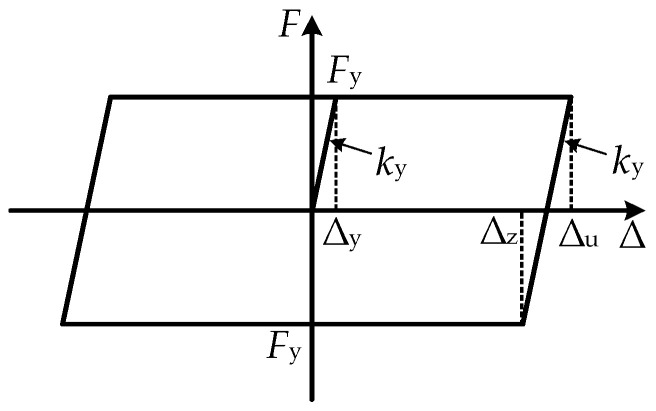
Theoretical restoring-force model of the X-shaped MS damper.

In order to facilitate the design, the ideal elasto-plastic constitutive model is adopted for the X-shaped energy-dissipation mild-steel material in this paper. According to the literature [34], the calculation formulas of the yield force *F*_y_, the yield displacement ∆_y_, the ∆_z_ of loading and unloading, and the stiffness *k*_y_ of a single X-shaped mild steel can be known as Equations (8)–(11):

(8) $$F_{y}=\frac{\sigma_{y}bt^{2}}{3h}$$

(9) $$\Delta_{y}=\frac{\sigma_{y}h^{2}}{2Et}$$

(10) $$k_{y}=\frac{F_{y}}{\Delta_{y}}=\frac{2E_{y}bt^{3}}{3h^{3}}$$

(11) $$\Delta_{z}=\Delta_{u}-2\Delta_{y}$$

where *b*, *h,* and *t* are the width, height, and thickness of a piece of X-shaped mild steel, respectively; *σ_y_* and *E_y_* are the yield stress and elastic modulus of the material, respectively. The restoring-force model of the MS energy-dissipation system, as derived using the theoretical formula above, is illustrated in Figure 8.

### 3.3. Restoring-Force Model of the MS-SCFD

**Figure 9:**
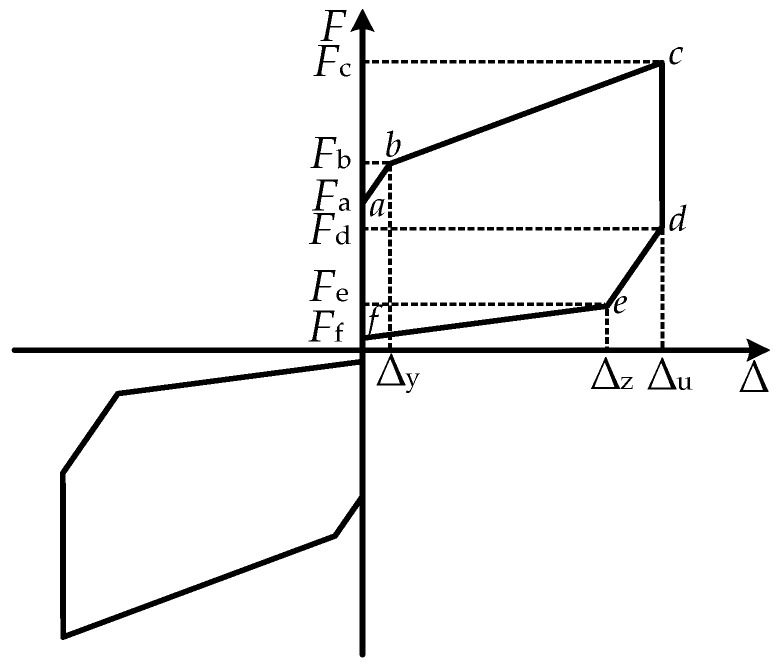
Theoretical restoring-force model of the MS-SCFD.

This paper assumes that, apart from the deformation of the combined disc springs and X-shaped energy-dissipation mild steel, all other components are considered rigid bodies without any deformations. The restoring-force model of the MS-SCFD can be expressed as a linear superposition of the restoring-force models for both the MS and the SCFD systems. The MS-SCFD as a whole exhibits an ideal flag-shaped hysteretic behavior, as illustrated in Figure 9.

The key performance points (from a to f) in the loading and unloading operation of the MS-SCFD are computed using the following equations:

(12) $$F_{a}=F_{\mathrm{ts}}$$

(13) $$F_{b}=F_{\mathrm{ts}}+2F_{y}$$

(14) $$F_{c}=F_{\mathrm{tu}}+2F_{y}$$

(15) $$F_{d}=F_{\mathrm{tr}}+2F_{y}$$

(16) $$F_{e}=F_{\mathrm{tr}}-2F_{y}$$

(17) $$F_{f}=F_{\mathrm{tc}}-2F_{y}$$

where *F*_a_ is the force of the MS-SCFD when the SCFD energy-dissipation system starts slipping; *F*_b_ and *F*_c_ are the forces when the displacement is loaded to the X-shaped mild-steel yield displacement ∆_y_ and the maximum displacement ∆_u_ during the loading process, respectively; *F*_d_ is the unloading instantaneous force for the MS-SCFD; *F*_e_ and *F*_f_ are the forces when the MS-SCFD is unloaded to the displacement ∆_z_ and restored to the initial equilibrium position, respectively.

In order to effectively achieve the self-centering function of the MS-SCFD, the initial compression amount of the combined disc spring should generate a restored force that is greater than or equal to the yield load of the X-shaped system. The relationship equation is as follows:

(18) $$F_{\mathrm{tc}}\geq 2F_{y}$$

## 4. Finite Element Modeling and Result Verification

### 4.1. Numerical Model of the MS-SCFD

The MS-SCFD utilizes LY160 steel as X-shaped energy-dissipation mild steel and Q690 steel for other components. The mechanical property parameters of each material are shown in Table 1. The combined disc springs consist of series A disc springs, featuring an inner diameter measuring 102 mm and an outer diameter measuring 200 mm. The superposition mode is a single-layer involution. The initial pre-pressure is 200 kN, and the stiffness is 55.2 kN/mm. Table 2 displays the dimensions of each significant damper component. Two analysis conditions were established to investigate the enhancement effect of X-shaped mild steel on the mechanical properties of dampers. Condition 1 was the SCFD without the X-shaped energy dissipation mild steel, and condition 2 was the complete MS-SCFD.

**Figure 10:**
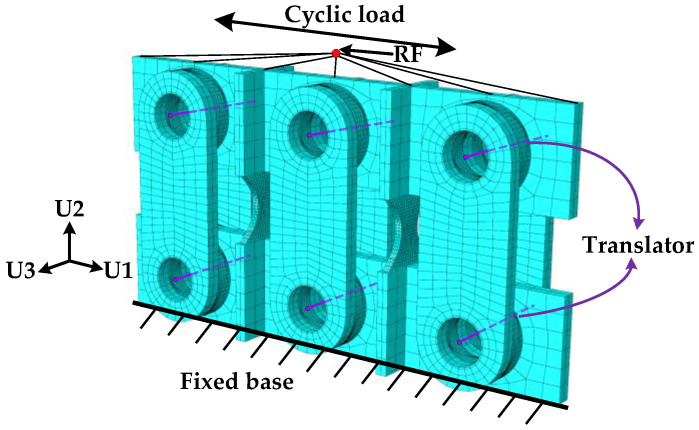
FE model of the MS-SCFD.

In order to verify the accuracy of the MS-SCFD theoretical restoring-force model and numerical model, the geometric models of the inner and outer friction plates were first established using the APDL language based on the finite element software ANSYS 17.0, and the fine models of the friction plates were established using the swept volumetric mesh technique combined with the Boolean operation, and then imported into ABAQUS 2023 for calculation. Ideal elasto-plastic constitutive models characterized the X-shaped energy dissipation of mild steel and other components in the MS-SCFD, while the C3D8R hexahedral reduced integration solid elements were employed for all component element types. The mesh refinement degree of the friction plate and mild steel is set to 10 mm in order to enhance simulation accuracy and calculation efficiency, while the dimensions of other components are approximately estimated at around 50 mm. Both the “Hard contact” normal behavior and the “Penalty function” tangential behavior with a friction coefficient of 0.2 were taken into consideration for the contact modeling between the inner and outer friction plates. Each pin hinge position was simulated using a hinge connection unit. The combined disc springs were simulated using the translator, while the pre-pressure of the combined disc springs was simulated using the setting mode of reference length. The finite element model of the MS-SCFD is shown in Figure 10. The center point at the top of the middle plate is set as the reference point RF, which is coupled with the upper surface of the middle plate for motion, and the load is applied to RF to simulate the low-cycle reciprocating loading process of the middle plate. During the loading process, the displacement control loading mode is adopted. The initial loading displacement is 10 mm, and each subsequent increment is also 10 mm. Each stage undergoes two loading cycles, with the loading ending at 40 mm.

### 4.2. Verification of the Theoretical Restoring Model

**Figure 11:**
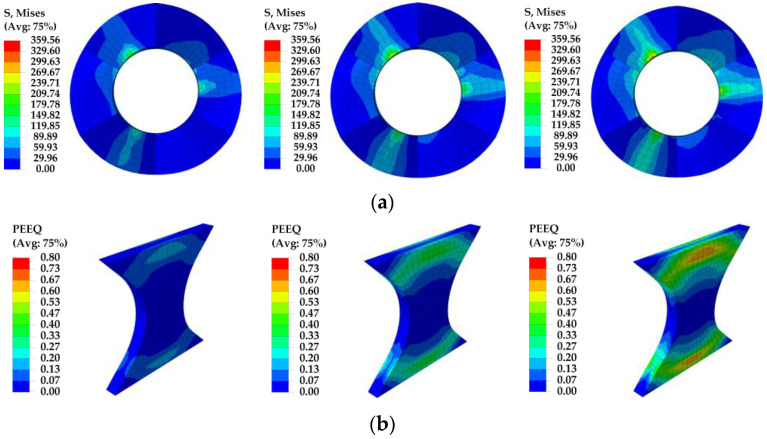
Simulation results of the MS-SCFD model. (**a**) Inner friction plate; (**b**) X-shaped mild steel.

Figure 11 depicts the von Mises stress contour of the inner friction plates and equivalent plastic strain (PEEQ) of the X-shaped mild steel for the MS-SCFD loading amplitude of 20 mm, 30 mm, and 40 mm. It can be observed that the stress was uniformly distributed on the operative helical friction surface and increased gradually with the increment of displacement amplitude. When the displacement reached 40 mm, the maximum stress was 359.6 MPa, which was significantly lower than the yield strength of the material of 690 MPa. The plastic deformation of X-shaped energy-dissipation mild steel majorly occurred at both ends of the steel plates, and the middle part remained elastic without any yield deformation. The simulation results demonstrate that the force and deformation characteristics align precisely with the design expectations. After damaging the dampers, only the X-shaped mild steels need to be replaced, while the friction plates are reusable.

**Figure 12:**
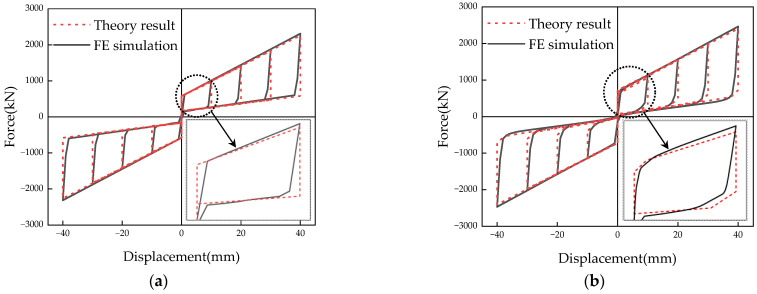
Simulation results versus theory results of the MS-SCFD and the SCFD. (**a**) SCFD; (**b**) MS-SCFD.

The hysteresis curves of the SCFD and MS-SCFD from the theoretical calculations and numerical simulation in ABAQUS 2023 are shown in Figure 12a,b, respectively. It can be observed from the figure that the hysteresis curves of the SCFD and the MS-SCFD obtained through theoretical calculations are essentially consistent with the results simulated using ABAQUS 2023, both exhibiting pronounced flag-shaped hysteresis characteristics. Table 3 shows the comparison results of the bearing capacity values of the key performance points of the dampers when the two dampers are loaded to 10 mm and 40 mm. The absolute values of the maximum errors in key performance points on the hysteresis curves of the SCFD and the MS-SCFD occur at the bearing capacity values of 6.83% and 9.46%, respectively. Furthermore, the error of other performance points is below 6.5%. It can be inferred that the theoretical restoring-force models have sufficient accuracy, which is capable of accurately describing the hysteretic performance of the dampers.

### 4.3. Comparison of Hysteresis Curves between the MS-SCFD and the SCFD

**Figure 13:**
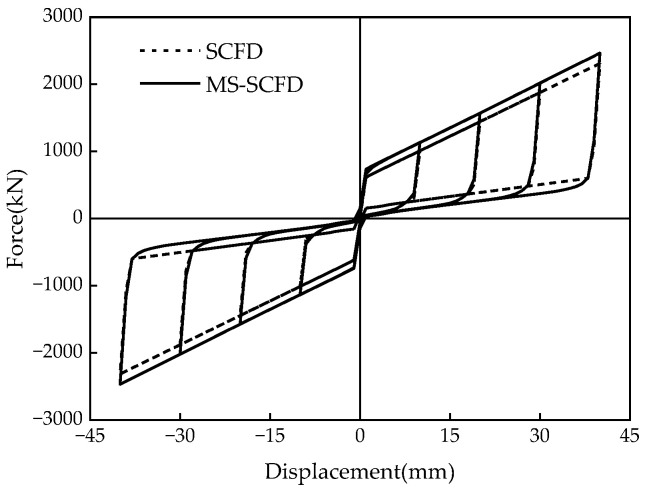
Comparison of hysteresis curves between the MS-SCFD and the SCFD.

As can be seen from Figure 13, the hysteresis curve of the MS-SCFD appears plumper compared to that of the SCFD damper, while its shape remains essentially unchanged. There is no significant difference in the initial stiffness between the two dampers, and their post-yield stiffness is identical. This can be attributed to utilizing an X-shaped mild steel material in this study, which serves as an ideal elasto-plastic model with a post-yield stiffness of 0. Additionally, the MS-SCFD exhibited a 6.6% increase in ultimate bearing capacity compared to the SCFD, indicating a modest enhancement. This suggests that the utilization of the X-shaped mild steel with a slightly lower bending yield load ensures optimal performance and complete self-centering behavior of the MS-SCFD. The observation reveals that the self-centering performance of the MS-SCFD remains unchanged as its bearing capacity increases, enabling it to return to its initial equilibrium position without any noticeable residual deformation.

**Figure 14:**
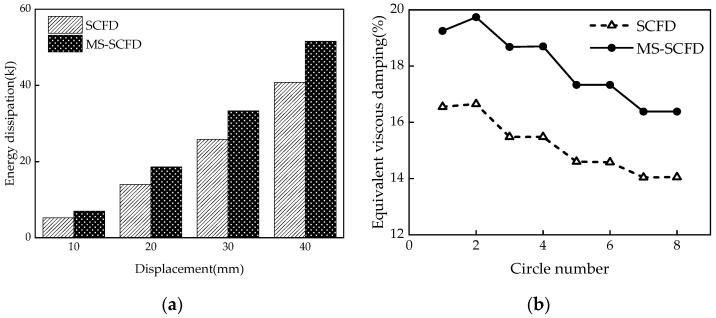
Comparison of the energy-dissipation capacity between the MS-SCFD and the SCFD. (**a**) Energy dissipation per cycle; (**b**) equivalent viscous damping ratio.

The energy dissipation per cycle and equivalent viscous damping ratios ζ_eq_ of the MS-SCFD and SCFD for different displacement amplitudes are shown in Figure 14a,b. Compared with the SCFD, when the displacement is loaded to 20 mm, 30 mm, and 40 mm, the energy dissipation per cycle of the MS-SCFD increases by 32.9%, 29.1%, and 26.6%, respectively. It is evident that under minor deformation, the energy dissipation of the MS-SCFD grows more considerably, which is advantageous for the damper’s ability to dissipate energy at low displacement levels. Compared to the SCFD damper, it enhances the equivalent viscous damping ratio of the MS-SCFD damper. Under a displacement load of 20 mm, the SCFD damper exhibits an equivalent viscous damping ratio of 0.167, whereas the MS-SCFD damper demonstrates an increased value of 0.198, representing an enhancement of 18.6%. These findings indicate that while maintaining the inherent self-centering advantage of the SCFD, the MS-SCFD also substantially improves energy-dissipation performance. The MS-SCFD proposed in this study is akin to that of the self-centering damper put forward by Chen et al. [35]. The damper, a hybrid energy-dissipation self-centering device, combines a buckling-restrained brace and ring spring friction. Both dampers use friction and metal yield as dual energy-dissipation mechanisms and necessitate an initial pre-pressure in the self-centering system that surpasses the yield load of the energy-dissipation metal materials to ensure the complete restoration of the dampers.

## 5. Parameter Analysis

Three sets of seven numerical models were designed for comparative analysis to investigate the influence of different parameters on the mechanical properties of the damper. The primary considerations were the influences of the disc spring pre-pressure, the friction coefficient, and the thickness of the X-shaped mild steel on the mechanical properties. The specific design conditions are summarized in Table 4.

### 5.1. Disc Spring Pre-Pressure

**Figure 15:**
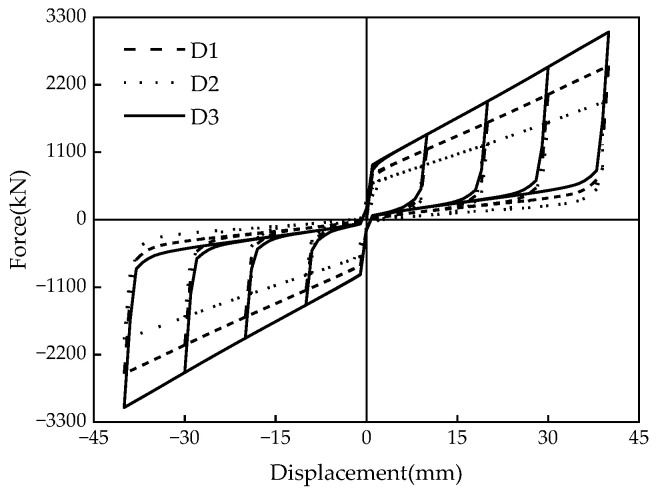
Hysteresis curves of the MS-SCFD under different *P*.

Figure 15 illustrates how the variations of the disc spring pre-pressure *P* (i.e., 150 kN, 200 kN, and 250 kN) affect the hysteretic behavior of the MS-SCFD. With the increase in *P*, both the initial stiffness and post-yield stiffness of the MS-SCFD are significantly enhanced. Additionally, the slipping force and ultimate force experience a substantial increase while the hysteresis curve becomes plumper. The residual deformation after unloading reached a maximum of 2.81 mm, 0.87 mm, and 0.74 mm, respectively. The self-centering performance of the MS-SCFD has undergone significant enhancements.

**Figure 16:**
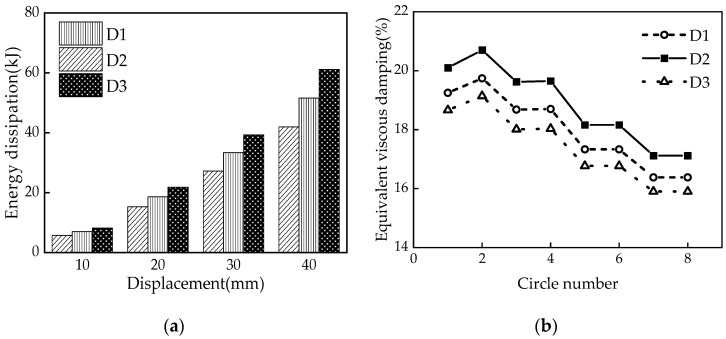
Energy-dissipation capacity of the MS-SCFD under different *P.* (**a**) Energy dissipation per cycle; (**b**) equivalent viscous damping ratio.

The changes in per cycle energy dissipation and ζeq of the MS-SCFD under different initial pre-pressures are illustrated in Figure 16. Figure 16a demonstrates that as the displacement amplitude increases, there is a gradual increase in the energy dissipation per cycle for each condition. When loaded to the maximum displacement of 40 mm, compared with the pre-pressure *P* = 150 kN, the damper with a pre-pressure of *P* = 250 kN increases energy dissipation by 45.8% per cycle. However, the equivalent viscous damping ratio ζeq decreases by 7.7%, and there is a slight reduction in the energy dissipation efficiency of the damper.

### 5.2. Friction Coefficient

**Figure 17:**
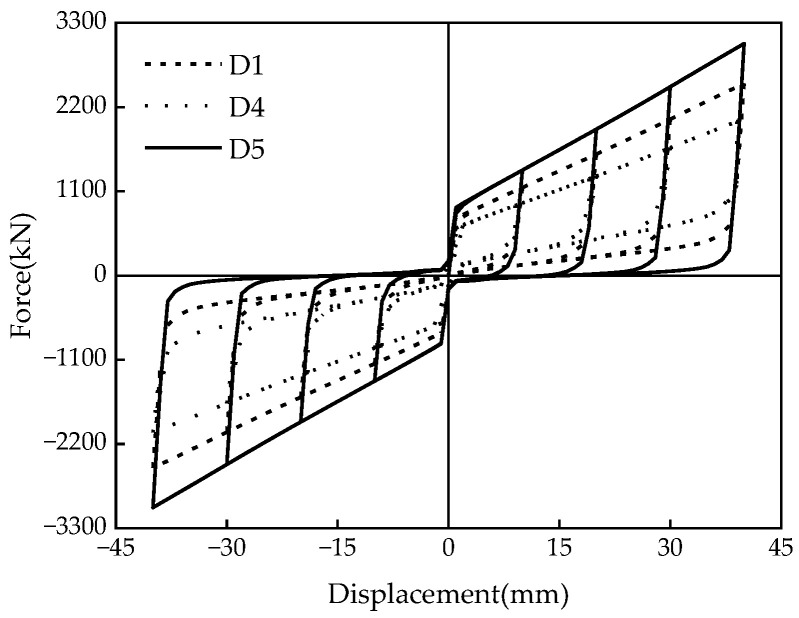
Hysteresis curves of the MS-SCFD under different *μ*.

The friction coefficient *μ* directly affects the mechanical properties of the MS-SCFD. Hysteresis curves of the damper with different friction coefficients (i.e., 0.1, 0.2, and 0.3) are shown in Figure 17. The friction coefficient significantly influences the slipping force, the ultimate force, the loading stiffness, and the energy-dissipation capacity. With an increase in the friction coefficient, both the slipping and ultimate forces of the damper exhibit a significant rise. The growth trend of ultimate bearing capacity slightly surpasses that of the slipping force. When *μ* is increased from 0.1 to 0.3, the ultimate force of the damper increases from 2047.7 kN to 3028.8 kN; however, there is a reduction in restored force. The maximum residual deformation increases from 0.47 mm to 15.9 mm, indicating a decrease in self-centering performance.

**Figure 18:**
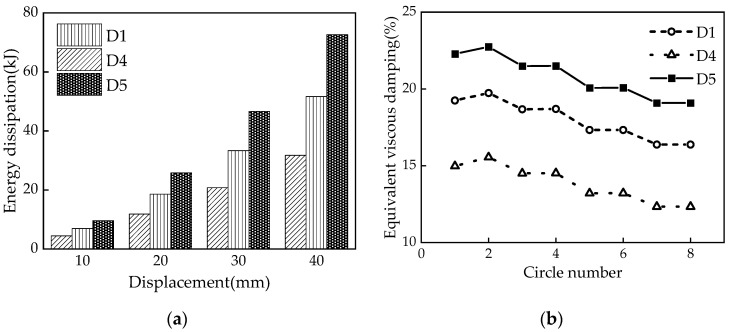
Energy-dissipation capacity of the MS-SCFD under different *μ.* (**a**) Energy dissipation per cycle; (**b**) equivalent viscous damping ratio.

According to Figure 18, as *μ* increases from 0.1 to 0.3, there is a corresponding increase in the energy dissipation per cycle at maximum displacement from 31.8 kJ to 72.7 kJ. ζ_eq_ also rises from 0.123 to 0.191, exhibiting a maximum rate of increase of 55.3%. This demonstrates that increasing the friction coefficient can effectively enhance the damper’s energy-dissipation capacity and efficiency.

### 5.3. Mild Steel Thickness

**Figure 19:**
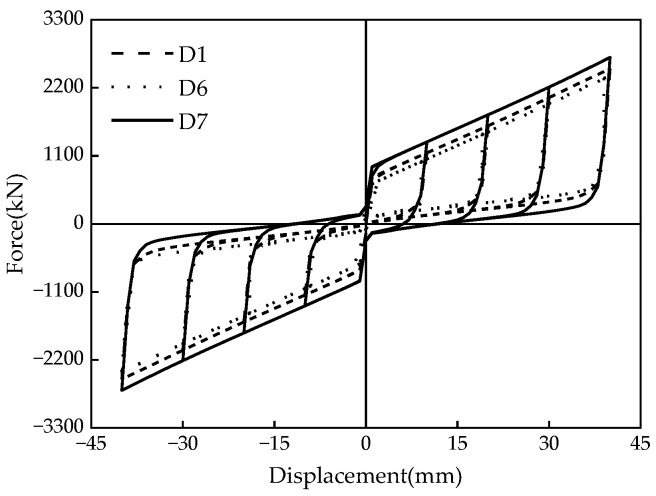
Hysteresis curve of the MS-SCFD under different *t*.

According to Figure 19 and the aforementioned theories, increasing the mild steel thickness (i.e., 15 mm, 30 mm, and 45 mm) effectively enhances the damper’s initial stiffness, the slipping force, and the ultimate force; however, it does not alter the post-yield stiffness of the damper. The restored force and the unloading instantaneous force of the MS-SCFD also diminish as the mild steel thickness increases. The overall hysteresis curves of the MS-SCFD exhibit a longitudinal expanding tendency, demonstrating the linear superposition of the MS system and the SCFD system in the mechanical properties of the MS-SCFD. The residual deformation of the MS-SCFD after unloading is 0.39 mm, 0.87 mm, and 11.97 mm, respectively. The main reason for the change in residual deformation is an increase in the mild steel thickness, which lowers the reset ratio and causes the damper’s residual deformation to rise. In order to satisfy the structural criteria for seismic design and ensure resilient performance, the reset ratio, bearing capacity, and energy-dissipation demands should all be carefully taken into account when designing the MS-SCFD.

**Figure 20:**
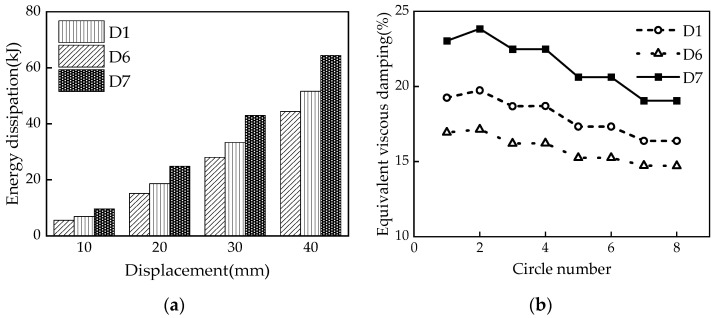
Energy-dissipation capacity of the MS-SCFD under different *t.* (**a**) Energy dissipation per cycle; (**b**) equivalent viscous damping ratio.

The energy dissipation per cycle and ζeq of the MS-SCFD exhibit variations with the increase in mild steel thickness, as illustrated in Figure 20. Figure 20a shows that the energy dissipation per cycle at the maximum displacement increases by 16.3% and 45.1%, respectively, when the mild steel thickness increases from 15 mm to 30 mm and 45 mm. Therefore, it can be reasonably expected that the energy-dissipation performance is significantly improved. Simultaneously, an increase in the thickness of the X-shaped mild steel leads to a more noticeable increase in ζeq. In Figure 20b, an increase in ζeq from 0.171 to 0.238 is observed as the mild steel thickness increases from 15 mm to 45 mm. The increase in mild steel thickness is observed to enhance the overall energy-dissipation level of the MS-SCFD significantly. Only replacing the X-shaped energy-dissipation mild steel can achieve the overall energy-dissipation enhancement of the damper without replacing the components of the SCFD system.

## 6. Conclusions

A hybrid self-centering damper incorporating both a friction and a metal yield energy-dissipation mechanism (MS-SCFD) is proposed. Its basic structure and working mechanism were introduced. A new theoretical restoring force model describing its hysteresis behavior was established. The numerical model of the new damper was simulated on the ABAQUS 2023 with low-cycle reciprocating loading tests. The following are the primary conclusions:

1. The damper makes up for the limitation of traditional metal yield dampers that cannot dissipate energy at the linear elastic stage with low displacement. Additionally, by incorporating the X-shaped mild steel for energy dissipation, the SCFD can effectively enhance its energy-dissipation performance, addressing the issue of a single energy-dissipation mechanism and insufficient energy consumption in traditional self-centering dampers;
2. The hysteresis curve of the MS-SCFD is smooth and plump, exhibiting typical flag characteristics and excellent self-centering performance. The simulation results of the numerical model are essentially consistent with the theoretical results, with a maximum error at crucial performance points of bearing capacity being only 9.46%;
3. The equivalent viscous damping coefficient and accumulated energy dissipation of the MS-SCFD can be effectively enhanced by increasing either friction coefficient or mild steel thickness; however, these measures lead to a slight decrease in self-centering performance. On the other hand, raising the pre-pressure on combined disc springs improves the self-centering ability while slightly reducing the energy-dissipation efficiency. Factors such as reset ratio, bearing capacity, and energy dissipation must be comprehensively considered to meet structural seismic performance requirements.

The mechanical properties of the MS-SCFD proposed in this study have only been preliminarily examined via theoretical analysis and numerical simulation. The theoretical calculation process encompasses rigid body assumptions and approximate relationships, while the numerical simulation process employs a relatively simple ideal elasto-plastic constitutive model for LY160 mild steel. Both results necessitate further model tests.

## Figures and Tables

**Table 1:** Constitutive model of the MS-SCFD.

Types of Material Parameters	Non-X-Shaped Mild Steel Parts	X-Shaped Mild Steel
Young’s elastic modulus (Gpa)	210	203
Yield strength (Mpa)	690	160
Tensile strength (Mpa)	860	-
Poisson’s ratio	0.294	0.3

**Table 2:** Key component parameters of the MS-SCFD.

Parameter Type	*R*_in_	*R*_out_	*H*	*L*	*b*	*h*	*t*
Data (mm)	102	200	56.1	748	300	306	30

**Table 3:** Calculation results of key performance points of the MS-SCFD and the SCFD.

Damper Category		SCFD				MS-SCFD					
		*F*_ts_	*F*_tu_	*F*_tr_	*F*_tc_	*F*_a_	*F*_b_	F_c_	F_d_	F_e_	F_f_
∆ = 10 mm	Simulation results (kN)	615.0	1010.1	247.1	156.7	614.8	719.8	1134.3	352.7	109.8	68.9
	Theoretical results (kN)	579.8	971.0	249.5	168.2	579.8	724.0	1065.1	343.6	103.3	74.1
	Error (%)	6.07	4.02	−0.93	−6.83	6.03	−0.58	6.50	2.65	6.29	−7.02
∆ = 40 mm	Simulation results (kN)	614.8	2312.7	600.3	156.8	615.5	719.8	2465.4	673.9	145.1	67.1
	Theoretical results (kN)	579.8	2262.8	581.3	168.2	579.8	724.0	2404.0	649.7	154.3	74.1
	Error (%)	6.03	2.21	3.27	−6.79	6.16	−0.58	2.55	3.59	−5.96	−9.46

**Table 4:** Design of conditions.

Model Definition	Model Number	*P* (kN)	*μ*	*t* (mm)
Benchmark model	D1	200	0.2	30
Pre-pressure change	D2	150	0.2	30
	D3	250	0.2	30
Friction coefficient change	D4	200	0.1	30
	D5	200	0.3	30
Mild steel thickness change	D6	200	0.2	15
	D7	200	0.2	45

## Data Availability

The data presented in this study are available upon request from the corresponding author.
